# Bacteriological characteristics of primary breast abscesses in patients from the community in the era of microbial resistance

**DOI:** 10.61622/rbgo/2024rbgo34

**Published:** 2024-04-09

**Authors:** Vicente Sperb Antonello, Jessica Dallé, Mirela Foresti Jimenez, Patrícia Tramontini, Andrei Gustavo Reginatto

**Affiliations:** 1 Hospital Fêmina Porto Alegre RS Brazil Hospital Fêmina, Porto Alegre, RS, Brazil.

**Keywords:** Abscess;, Breast diseases, Microbial drug resistance, Staphylococcus aureus, Sulfamethoxazole

## Abstract

**Objective:**

The aim of this study is to evaluate the etiological profile and antimicrobial resistance in breast abscess cultures from patients from the community, treated at a public hospital located in Porto Alegre, Brazil.

**Methods:**

This is an retrospective cross-sectional study that evaluated the medical records of patients with bacterial isolates in breast abscess secretion cultures and their antibiograms, from January 2010 to August 2022.

**Results:**

Based on 129 positive cultures from women from the community diagnosed with breast abscesses and treated at Fêmina Hospital, 99 (76.7%) of the patients had positive cultures for *Staphylococcus sp*, 91 (92%) of which were cases of *Staphylococcus aureus*. Regarding the resistance profile of *S. aureus*, 32% of the strains were resistant to clindamycin, 26% to oxacillin and 5% to trimethoprim-sulfamethoxazole. The antimicrobials vancomycin, linezolid and tigecycline did not show resistance for *S. aureus*.

**Conclusion:**

*Staphylococcus aureus* was the most common pathogen found in the breast abscess isolates during the study period. Oxacillin remains a good option for hospitalized patients. The use of sulfamethoxazole plus trimethoprim should be considered as a good option for use at home, due to its low bacterial resistance, effectiveness and low cost.

## Introduction

A breast abscess is defined as an organized collection from an exudative inflammatory process that affects the breast tissue secondary to an untreated mastitis or that does not respond to the initial antimicrobial regimen. It is a significant cause of mortality due to the functional incapacity it causes in these patients and its high rate of recurrence.^(1)^ The main risk factor for the formation of breast abscesses is mastitis. Other associated risk factors are lactation, obesity, and smoking, the latter of which is the only one related with recurrence of the pathology.^(2)^

In relation to its microbiology, it is known that the main pathogen related to the disease is *Staphylococcus aureus*. Also, there has been a perceived increase in the prevalence of *Staphylococcus aureus* that is resistant to methicillin (MRSA) originating from the community.^(3)^ Other less commonly associated pathogens include *Streptococcus pyogenes, Escherichia coli, Bacteroides species, Corynebacterium species*, coagulase-negative *Staphylococcus* (*S. lugdunensis*), *Pseudomonas aeruginosa, Proteus mirabilis* and anaerobes.^(4)^ Patients with recurrent abscesses present a mixed flora associated with a greater prevalence of anaerobes and a lower prevalence of *S. aureus* and MRSA.^(2,5)^ Infection by MRSA is more common in patients who have recently undergone hospitalization or surgery, hemodialysis, have human immunodeficiency virus (HIV), use intravenous drugs, or have previously used antibiotics.^(1)^

The aim of this study is to assess the etiological and antimicrobial sensitivity profile in breast abscess secretion cultures from patients originating from the community, treated by the Department of Gynecology and Obstetrics of Hospital Fêmina, which belongs to the Conceição Hospital Group, in Porto Alegre, Brazil.

## Methods

A retrospective cross-sectional study was conducted with results from breast abscess secretion cultures collected from patients originating from the community and treated at referal hospital. We retrieved and reviewed medical records of patients with a diagnosis of breast abscess who were admitted to the Gynecology/Mastology Unit for specific treatment in the period from January of 2010 to August of 2022, with confidentiality of patient identification to all researchers. Informed consent was waived by the ethics committee, because all data was provided by medical records.

The cultures were identified based on weekly communication from the Microbiology Laboratory of the institution regarding positive microbiological results to the Infection Control Service of Fêmina Hospital and allocated daily in a database. The analysis was performed with IBM SPSS Statistics for Windows, Version 20 (IBM Corp, Armonk, NY, USA). Categorical variables were described by frequencies and percentages.

The inclusion criterion for the study was breast secretion cultures obtained from a patient from the community treated at Fêmina Hospital, collected up to 72 hours after admission.^(6)^ Patients admitted to Fêmina Hospital in the last 90 days were excluded. Individuals with recurrent abscess or disease after breast surgical intervention were excluded of the study. This was justified to the analysis of only primary breast abscess. This study was approved by the Research Ethics Committee (5.592.156) of the Conceição Hospital Group, under protocol number CAE 61086222.0.0000.5530 in August, 19 2022 and data was statistically analyzed from September to December 2022.

## Results

**Figure 1:**
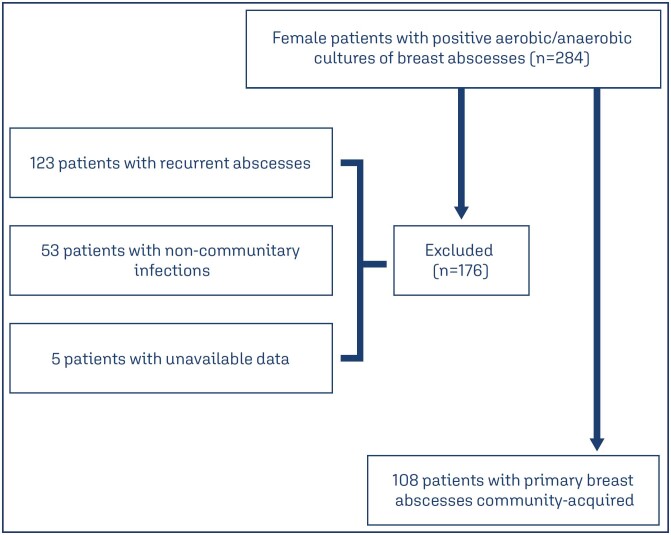
Flow diagram of included and excluded patients in the study

In 12 years, 108 female patients from the community were identified with positive aerobic/anaerobic cultures of breast abscesses. The mean age of the patients was 35.9 years (age range: 13 to 78 years) and there were 34 (31.5%) in the breastfeeding group, 27 (25%) were obese, 21 (19.4%) were smokers, 17 (15.7%) had diabetes, 15 (13.9%) had breast cancer, and 5 (4.6%) were HIV positive. Considering the 108 patients who underwent a diagnostic breast abscess puncture, there were 129 positive culture isolates, with 99 (76.7%) positive cases for *Staphylococcus sp*. Of these, 91 (92%) were *Staphylococcus aureus*, 5 (5%) were *Staphylococcus epidermidis*, 1 (1%) was *Staphylococcus warneri*, 1 (1%) was *Staphylococcus capitis*, and 1 (1%) was coagulase-negative *Staphylococcus*. In relation to the other germs prevalent in the cultures analyzed, we observed *Streptococcus agalactiae* (4.7%), *Escherichia coli* (3.9%), *Proteus mirabilis* (3.9%), *Klebsiella* (1.5%) and *Pseudomonas* (1.5%). With less that 1% prevalence were *Peptococcus species*, *Enterococcus species*, *Bacteroides species*, *Enterobacter cancerogenus* and *Candida* (Figure 1).

The data related to the resistance profile of *Staphylococcus aureus* are described in table 1. Of the total breast secretion cultures, there was no occurrence of multi-resistant germs: *Staphylococcus aureus* with intermediate resistance to vancomycin (VISA) or *Staphylococcus aureus* resistant to methicillin (VRSA), according to the CDC (Centers for Disease Control and Prevention) criterion. However, 24 (26%) analyzed cases of *Staphylococcus aureus* were resistant to methicillin (MRSA).

**Table 1:** *Staphylococcus aureus* susceptibility and resistant pattern

Antimicrobial	Susceptibility [number of isolates, %], Resistant [number of isolates, %]	
	S	R
Clindamycin	62(68.1)	29(31.9)
Gentamicin	90(98.9)	1(1.1)
Linezolid	91(100)	0(0)
Oxacillin	67(73.6)	24(26.4)
Sulfamethoxazole/trimethoprim	87(95.6)	4(4.4)
Ciprofloxacin	60(65.9)	31(34.1)
Tigecycline	91(100)	0(0)
Vancomycin	91(100)	0(0)

S – Susceptible; R – Resistant

Considering the resistance profile for clindamycin in *Staphylococcus aureus* isolates, there were 29 (31.9%) cases, while for sulfamethoxazole-trimethoprim, only 4 (4.4%) of the cases were resistant. The antimicrobials vancomycin, linezolid and tigecycline did not present any isolate with resistance for *Staphylococcus aureus*.

## Discussion

Breast abscesses are a major cause of morbidity among the women affected, with a high chance of recurrence and high rates of breast deformities. Quickly establishing their treatment is fundamental to avoid complications. The management of breast abscesses includes draining the abscess and sending the material for culture, associated with antibiotic therapy and analgesic support measures.^(7-10)^

With respect to the antibiotics to be empirically used, treatment should include specific therapy for gram-positive bacteria.^(5,7,11,12)^ According to the literature, gram positive bacteria present a greater prevalence in positive cultures. Among these, *Staphylococcus aureus* is the most prevalent germ, varying between 32.9% and 52.94% in some studies, followed by *Enterococcus sp* and *Streptococcus sp*.^(7,9)^ In the present study, we found a predominance of *S. aureus*, which was observed in 71% of the primary breast abscess cultures.

In relation to the resistance profile in the present study, for oxacillin, clindamycin and ciprofloxacin, rates higher than 20% were observed in *S. aureus* isolates. These antimicrobials are widely used in the empirical treatment of breast abscesses.^(5,13)^ Considering resistance to oxacillin, the literature describes a variation from 1% to 58%, in studies conducted in various countries around the world.^(4,5,8,13-17)^ In our study, the rate was 26%.

The resistance indicators for clindamycin and ciprofloxacin in the literature varied from 0 to 42% and 3 to 51%, respectively.^(5,15-18)^ In the present study, the authors found a resistance profile of 32% for clindamycin and 24% for ciprofloxacin.

The antimicrobial sulfamethoxazole/trimethoprim presented a resistance profile of lower than 5%, representing an important alternative in the treatment of breast abscesses, especially for outpatients with an uncomplicated infection. According to Moazzez et al.,^(5)^ based on the known effectiveness, resistance potential, cost, and patient adhesion, sulfamethoxazole/trimethoprim therapy for patients with breast abscesses is a reality.

In the present study, there were no cases of *Staphylococcus aureus* with intermediate resistance to vancomycin (VISA) or *Staphylococcus aureus* resistant to vancomycin (VRSA). In the literature, there are few studies that mention the percentage of multi-resistant germs in breast abscess cultures according to the CDC definition or based on the resistance profile. According to the literature, the incidence of VISA/VRSA is hard to estimate due to the scarce cases and the challenges related to laboratory detection.^(1)^

Several authors indicate that the first line medications for treating breast abscesses include amoxicillin plus clavulanate and flucloxacillin (unavailable in Brazil).^(11)^ In uncomplicated cases the use of cefalexin and clindamycin represent good options, especially in patients allergic to penicillin.^(1)^ If there is an increased risk for MRSA, the use of sulfamethoxazole plus trimethoprim or clindamycin should be considered.^(7,12)^

Thus, according to the results presented, the use of oxacillin in admitted patients can be indicated. The use of amoxicillin/clavulanate or cefuroxime can also be indicated as it has a spectrum for *S. aureus* similar to oxacillin.^(5,19,20)^ The resistance profile found for the antimicrobial sulfamethoxazole/trimethoprim should be valued for possible choices, especially in antibiotic therapy at home. In the present study, there was also an optimal profile of susceptibility to vancomycin, tigecycline, and linezolid; however, the use of these drugs is more restricted.

In relation to the limitations of the study, the fact that the study was conducted at a single center and the reduced number of culture isolates should be considered. Data were captured from positive isolates. Therefore, we cannot specify the number of abscesses that were drained and not sent for antimicrobial culture in the last 12 years. There must be awareness of the surgeon and continued medical education about the importance of collecting material for microbiological analysis. Our data should be assessed with caution and new studies and a follow up of this study would show the tendency of susceptibility for these and other antibiotics in clinical practice.

## Conclusion

Finally, it is fundamental for empirical antimicrobial therapy for breast infectious syndromes to be initiated according to the susceptibility patterns described in the literature in studies such as this one, and the results of cultures should always be quickly evaluated for targeted treatment of the pathogen involved. In our study, *Staphylococcus aureus* was the most common pathogen found in the breast abscess isolates. Oxacillin remains a good option for hospitalized patients. The use of sulfamethoxazole plus trimethoprim should be considered as a good option for use at home, due to its low bacterial resistance, effectiveness and low cost.
